# Improving Transmission Line Fault Diagnosis Based on EEMD and Power Spectral Entropy

**DOI:** 10.3390/e26090806

**Published:** 2024-09-21

**Authors:** Yuan-Bin Chen, Hui-Shan Cui, Chia-Wei Huang, Wei-Tai Hsu

**Affiliations:** Department of Electrical Engineering, Zhaoqing University, Zhaoqing 526060, China; 2022010105@zqu.edu.cn (Y.-B.C.); yangzhizhong@zqu.edu.cn (H.-S.C.); 2018013014@zqu.edu.cn (C.-W.H.)

**Keywords:** fault diagnosis, time series, power spectral entropy, transmission line

## Abstract

The fault diagnosis on a transmission line based on the characteristics of the power spectral entropy is proposed in this article. The data preprocessing for the experimental measurement is also introduced using the EEMD. The EEMD is used to preprocess experimental measurements, which are nonlinear and non-stationary fault signals, to overcome the mode mixing. This study focuses on the fault location detection of transmission lines during faults. The proposed method is adopted for different fault types through simulation under the fault point by collecting current and voltage signals at a distance from the fault point. An analysis and comprehensive evaluation of three-phase measured current and voltage signals at distinct fault locations is conducted. The form and position of the fault are distinguished directly and effectively, thereby significantly improving the transmission line efficiency and accuracy of fault diagnosis.

## 1. Introduction

The fault diagnosis technology is a fundamental guarantee for the safe application of the power system [1,2]. Time series analysis is the most important and commonly used method for signal processing in power system fault diagnosis. In practice, the measured signals vary with time, describing signal changes over time. Nevertheless, the circumstances and advancement of faults often cause changes in the time-frequency formation [3,4]. For example, when a fault occurs in the power system, the fault signal contains multiple transient signals. The transient component changes with time, as do the fault spot, the fault spot’s transition resistance, and the utilities’ operating conditions [5,6].

Moreover, faults in transmission lines are caused by periodic shocks, which are difficult to distinguish in time domain analysis. However, these faults have corresponded to frequency components in the transient signal. Therefore, it is necessary to analyze the frequency-domain signal to understand and observe the dynamic behavior of the object through the measured signal. The frequency-domain analysis is viewed in the frequency domain by converting information in the time domain. The essence of spectrum analysis lies in utilizing the Fourier transform to dismantle intricate time series into distinct harmonic components, enabling the examination of the signal’s frequency structure and the complicated relationships among amplitude, phase, power, energy, and frequency within each harmonic [7,8,9]. 

When a transmission line fails, a sudden voltage change appears at the fault point. Under the action of this sudden change in voltage, moving disturbance waves are introduced on the transmission line. These traveling disturbance waves contain rich information [10,11,12,13]. In addition, the fault signal contains many transient signals after the system malfunctions. These transient signals are involved in the information about the type and severity of the fault. Faults lead to changes in power quality parameters, such as power factor, voltage fluctuations, and voltage flickers. When the fault occurs, the proportion of energy in the frequency domain of the fault component is increased. The energy magnitude is helpful as the index to distinguish faults [14].

The time characteristics of traditional Fourier transform for nonlinear and non-stationary signals are too poor to satisfy fault detection. Because of this, the ensemble empirical mode decomposition (EEMD) is introduced into fault detection to analyze the transient and disturbance signals, overcome the mode mixing phenomenon, and precisely detect the transient and disturbance spots through the instantaneous frequency [5]. In recent years, research on EEMD has deepened, and scholars have explored it from multiple aspects, such as theory, algorithms, and applications. In terms of algorithm optimization, researchers have proposed various improvement methods, such as adaptive noise addition strategy, optimizing the decomposition, etc., to improve the decomposition efficiency and accuracy of EEMD. In terms of application, EEMD has been successfully applied to multiple practical cases and achieved significant results. Although EEMD has made considerable progress in numerous fields, some key issues still need to be addressed urgently. For example, how to determine the optimal noise addition strategy and decomposition times to balance the decomposition effect and computational cost and further improve the decomposition accuracy and stability of EEMD in complex signal environments [15,16,17,18].

The approach of information entropy was proposed in 1948 when Shannon studied probability theory. Information entropy is described as the incompressibility of a system or thing. When the uncertainty of the probability distribution of the system is more significant, the value of information entropy contributed by the system is also more enormous. When the uncertainty of the probability distribution from the system is smaller, the corresponding information entropy is smaller. Therefore, information entropy is suitable for fault diagnosis [5,19,20,21]. The concept of power spectral entropy (PSE), also known as spectral entropy, was first proposed by Roberto Livi et al. in 1985 and is mainly used to measure energy sharing in nonlinear large Hamiltonian systems. Subsequently, this concept gradually expanded to other fields, especially in time series analysis and signal processing. Spectral entropy is based on information entropy, which describes the complexity and uncertainty of a system by quantifying the uneven distribution of different frequency components in the power spectrum [22]. Its theoretical basis includes entropy theory, time series analysis, and signal processing [23]. Specifically, spectral entropy calculates the probability distribution of each frequency component in the power spectrum and uses the entropy formula to measure its uncertainty [24]. In recent years, spectral entropy has made significant progress in multiple fields. In signal processing, spectral entropy is used to analyze the complexity and uncertainty of signals, such as speech signal recognition, biological signal processing, etc. [25,26]. In system complexity analysis, spectral entropy measures the stability and degree of chaos of the system’s dynamic behavior. Although spectral entropy has demonstrated its superiority in multiple fields, there are still some problems and challenges. First, the calculation method of spectral entropy is susceptible to data and requires precise data processing and preprocessing. Second, interpreting and applying spectral entropy requires a deep theoretical foundation and rich practical experience. Finally, how to better combine spectral entropy with other analysis methods to improve the accuracy and reliability of analysis is also a problem that needs to be solved in the future [27]. 

This paper establishes a fault feature extraction based on the combination of EEMD and PSE to achieve effective feature extraction of fault signals of transmission lines. EEMD’s combination of adaptivity, noise handling, simplicity, and interpretability makes it a compelling choice for many signal processing applications. PSE provides a valuable tool for quantifying signal complexity, extracting features, and detecting changes in frequency distributions. Its non-parametric nature and sensitivity to variations make it applicable to various domains. Combining the advantages of the two methods improves the discrimination of fault types and the location of fault for the transmission line, which is better used than one method in practice. Analyzing the changes in PSE value is suitable for distinguishing the types and spots and boosting the fault diagnosis efficiency for the transmission line [28,29].

## 2. Method

### 2.1. Ensemble Empirical Mode Decomposition

When a uniformly distributed white noise background is incorporated into the signal, the various signal regions’ scales are naturally aligned with the corresponding scales associated with the background noise. Given that the additional white noise is evenly spread across the time-frequency space, this space comprises multiple components of different scales, partitioned by a filter bank. However, it’s worth noting that each test may yield noisy outcomes due to including both the signal and the additional white noise within each noise component. Fortunately, as the noise varies across independent tests, it cancels out when calculating the overall mean of a sufficient number of tests. In this case, the *i*-th observation signal is as follows [30,31]:

(1) $$x_{i}(t)=x(t)+w_{i}(t)$$

where *x*(*t*) is the original signal, and *w_i_*(*t*) is white noise. The procedure of EEMD is described as follows:

(a) The target data is added to the white noise sequence.
(b) The target data with added white noise is decomposed into intrinsic mode functions (IMFs). Each IMF is expressed as

(2) $$x_{i}(t)=\sum_{j=1}^{N}c_{i,j}(t)+r_{i,j}(t)$$

where *c_i_*_,*j*_(*t*) is the *j*-th IMF obtained by decomposing after adding white noise for the *i*-th time, and *r_i_*_,*j*_(*t*) is the residual function.

(c) A different white noise sequence is added each time and repeats steps (a) and (b).
(d) The mean of each IMF obtained by decomposition is taken as the final result defined as

(3) $$c_{j}(t)=\frac{1}{M}\sum_{i=1}^{M}c_{i,j}(t)$$

where *c_j_*(*t*) is the *j*-th IMF of the EEMD decomposition, *i* = 1, 2, ... *M*, and *j* = 1, 2, ... *N*.

EEMD processes the measurement signal and then reconstructs it by eliminating some IMFs as the input signal [5]. The purpose of signal preprocessing is to convert the signal into a form suitable for digital processing to reduce the difficulty of digital processing. Due to the nonlinear and non-stationary characteristics of fault signals, traditional linear analysis methods have their limitations. EEMD has advantages for this type of signal and significantly affects the signal preprocessing stage. Therefore, this paper adopts EEMD as the signal preprocessing step.

### 2.2. Power Spectral Entropy

Power spectral entropy measures the uncertainty or complexity of a signal in the frequency domain. It depends on the concept of Shannon entropy or information entropy in information theory. It regards the normalized power spectrum distribution of the signal as a probability distribution and then calculates its information entropy. The power spectral entropy is defined as [32,33]

(4) $$PSE=-\sum_{i=1}^{n}p_{i}\ln p_{i}$$

where n is the number of frequency points, and $p_{i}$ is the value of the signal’s power spectrum at the *i*-th frequency point. Its calculation formula is

(5) $$p_{i}=\frac{{\left|X_{n}(f)\right|}^{2}/n}{\sum_{i=1}^{n}{\left|X_{n}(f)\right|}^{2}/n}$$

where $X_{n}(f)$ is the Fourier transform for the time series *x*(*t*). After the EEMD procedure, the reconstructed signal is the input for the process of the PSE.

The power spectral entropy describes the spectral structure of the fault signal. If the fault is distributed uniformly over the entire frequency component, the fault signal is complex, and the degree of uncertainty is enormous. The fault signal for the transmission line’s PSE value represents the fluctuation energy classification at various frequencies. If the energy is more uniformly distributed at each frequency, the greater the signal uncertainty, the greater the PSE value. If the energy is more unevenly distributed at each frequency, the smaller the signal uncertainty, the smaller the PSE value [28,34,35]. 

## 3. Results

**Figure 1:**
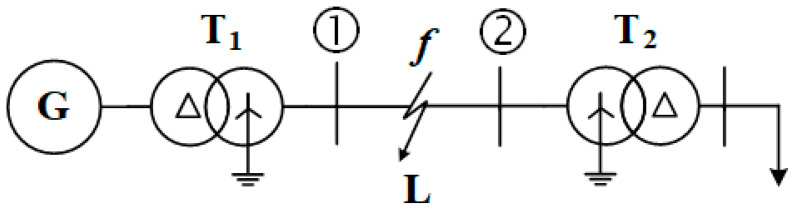
System diagram of the 110 kV power system.

The system diagram of the 110 kV power distribution is shown in Figure 1. A 50 Hz, 18.75 MVA, 10 kV power source (G), wye-connected generator, is connected through a step-up delta-wye grounded transformer (T_1_) to bus#1. Then, the transmission line (L = 100 km, LGJ-120) is connected to a step-down grounded wye-delta transformer (T_2_) through bus#2. The load (*P* = 3 MW, *Q* = 1.45 MVAR) is connected to the secondary side of T_2_. The three-phase rating of the transformer is 63 MVA, 10.5/110 kV (*V_s_*% = 10.5%, Δ*P_s_* = 234 kW, *I_o_*% = 0.48%, Δ*P_o_* = 52 kW). The fault (*f*) is simulated at the transmission line (L). The voltage and current signals of phases A, B, and C with a sample time of 1 × 10^−6^ are used in the study. The rated voltage is defined as 110 kV, and the rated capacity is set to 100 MVA. The 110 kV power distribution system is constructed in MATLAB/Simulink environment. The time duration is set to 0.2 s. The time duration of the fault is 0.05 s.

**Figure 2:**
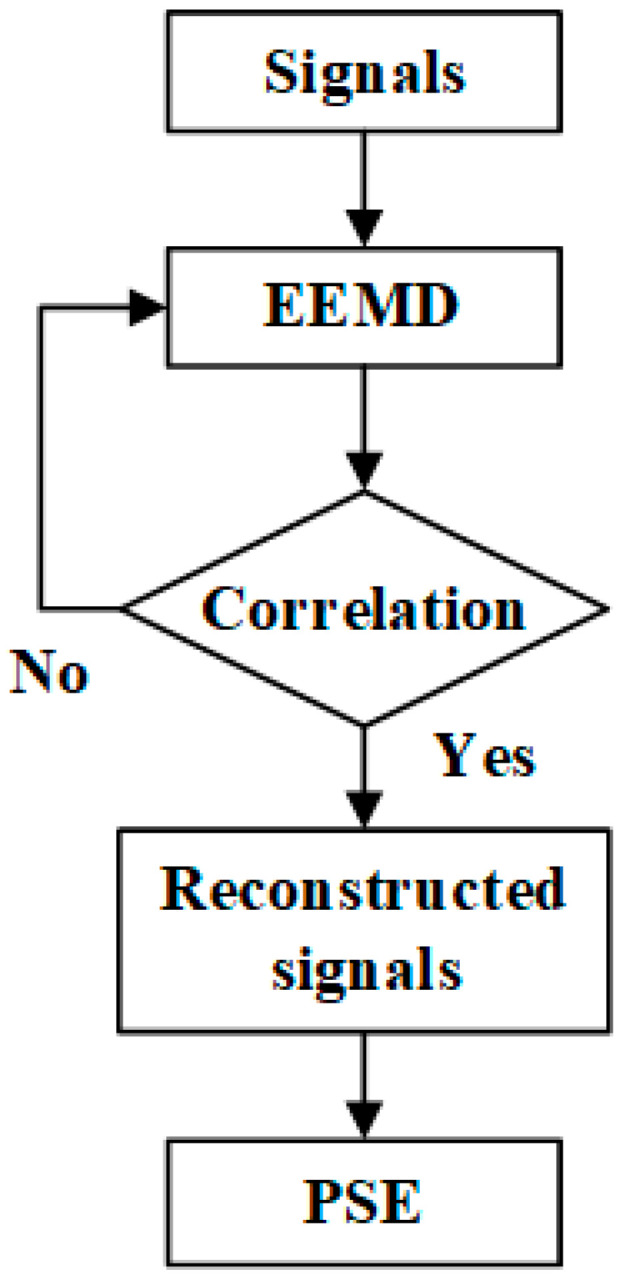
Flow chart of data processing.

When the fault occurs at different locations, such as ten segments (L), the three-phase current and voltage signal are collected through the measurement device at Bus#1. The various types of faults that happened on the 110 kV transmission line are analyzed, such as single line-to-ground fault (LG), double line-to-line fault (LL), double line-to-ground fault (LLG), and triple line-to-line fault (LLL). The three-phase current and voltage signal for regular operation (RO) is also collected. The flow chart of data processing is shown in Figure 2. The three-phase measured current and voltage signals are processed using the EEMD method. Then, the similarity analysis is used between signals and reconstructed signals by eliminating parts of IMFs. The similarity analysis aims to find the reconstructed signal processed by EEMD, whose waveform is close to the original signal. This article uses the correlation coefficient to compare the reconstructed and original signals. The final step for the reconstructed signal is to calculate the entropy value through power spectral entropy.

### 3.1. LG Fault

For the LG fault, the result of PSE for current and voltage data is shown in Table 1. The values of PSE for current and voltage during regular operation are smaller than those during fault conditions. The value of the PSE for the current signal (*i_A_*) is larger than the value of the other two signals (*i_B_* and *i_C_*), and the value of the PSE for the voltage signal (*v_A_*) is more significant than that of the other two signals (*v_B_* and *v_C_*). The enormous value of PSE for phase A indicates that the fault occurred in phase A. The value of PSE for the current signal (*i_A_*) is decreased as the distance from the fault location increases, and the difference in the current between the fault point and regular operation is also large. The value of the PSE for the voltage signal (*v_A_*) is decreased as the distance from the fault location increases.

### 3.2. LL Fault

For the LL fault, the result of PSE for current and voltage data is shown in Table 2. The values of the PSE for current and voltage signals during regular operation are smaller than those during fault conditions. The values of the PSE for current (*i_A_* and *i_B_*) and voltage signals (*v_A_* and *v_B_*) are larger than the value of another signal (*i_C_* or *v_C_*). The value of the PSE for current signals (*i_A_* and *i_B_*) drops as the distance from the fault location rises, and the difference between the fault point and regular operation is also enormous. The value of the PSE for voltage signals (*v_A_* and *v_B_*) is reduced as the distance from the fault location expands. The difference in the voltage between the fault point and regular operation is also significant.

### 3.3. LLG Fault

For the LLG fault, the result of PSE for current and voltage data is shown in Table 3. The values of the PSE for current and voltage signals during regular operation are smaller than those during fault conditions. The values of the PSE for current signals and voltage signals are raised. The value of the PSE for current signals (*i_A_* and *i_B_*) slowly diminishes as the distance from the fault location is aggravated, and the difference between the fault point and regular operation is also significant. The value of the PSE for voltage signals (*v_A_* and *v_B_*) is weakened as the distance from the fault location develops. The difference between the fault point and regular operation is also massive.

### 3.4. LLL Fault

For the LLL fault, the value of PSE for current and voltage data is shown in Table 4. The values of the PSE for three-phase signals of current and voltage during regular operation are smaller than those during the fault. The values of the PSE for current and voltage signals are raised. The value of the PSE for current and voltage signals almost ceases slowly as the distance from the fault location enlarges, and the difference between the fault point and regular operation is also significant.

**Figure 3:**
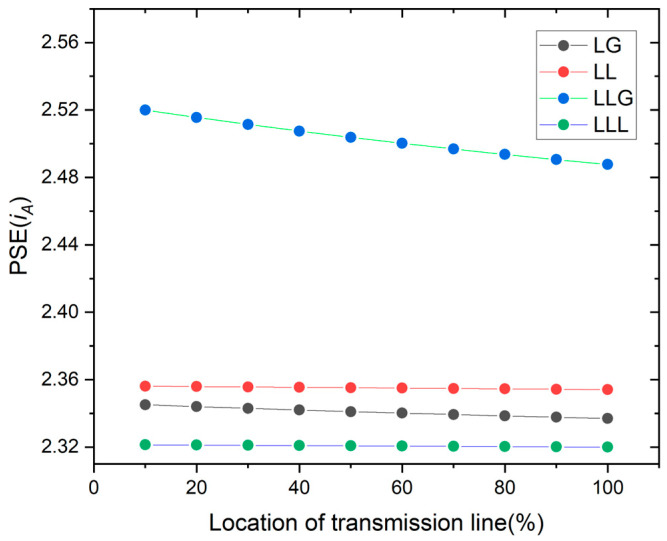
PSE of the current for all kinds of faults.

Figure 3 shows the PSE of the faulty current phase (*i_A_*). The fault location of the transmission line is observed by using the current value of the PSE for different types of faults. The location of the fault point is estimated using the percentages corresponding to different PSE values for phase A current.

**Figure 4:**
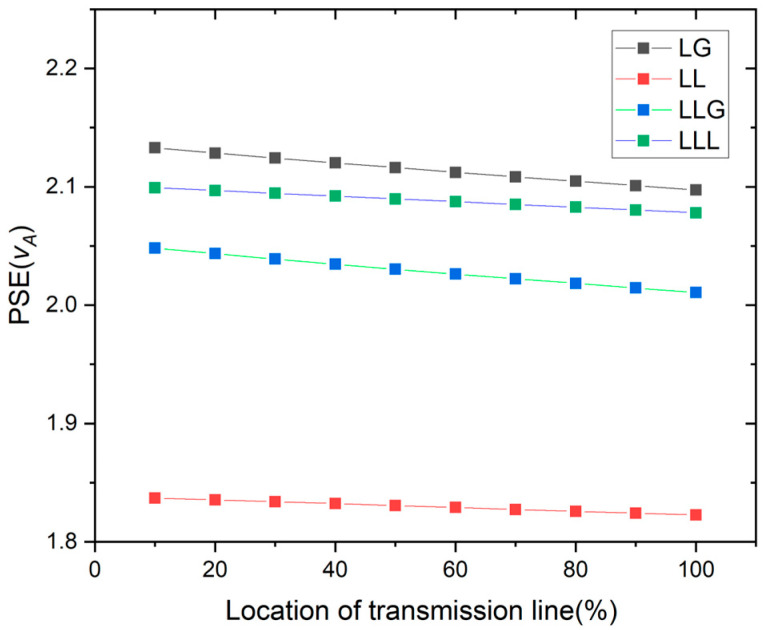
PSE of the voltage for all kinds of faults.

Figure 4 shows the PSE of the faulty voltage phase (*v_A_*). The fault location of the transmission line is recognized by using the voltage value of the PSE for different types of faults. The location of the fault point is estimated using the percentages corresponding to different PSE values for phase A voltage.

**Figure 5:**
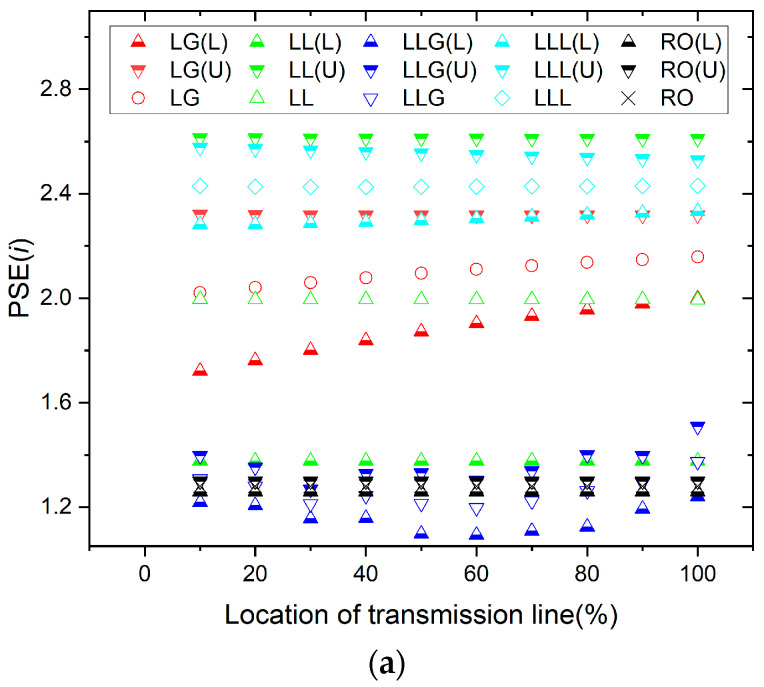

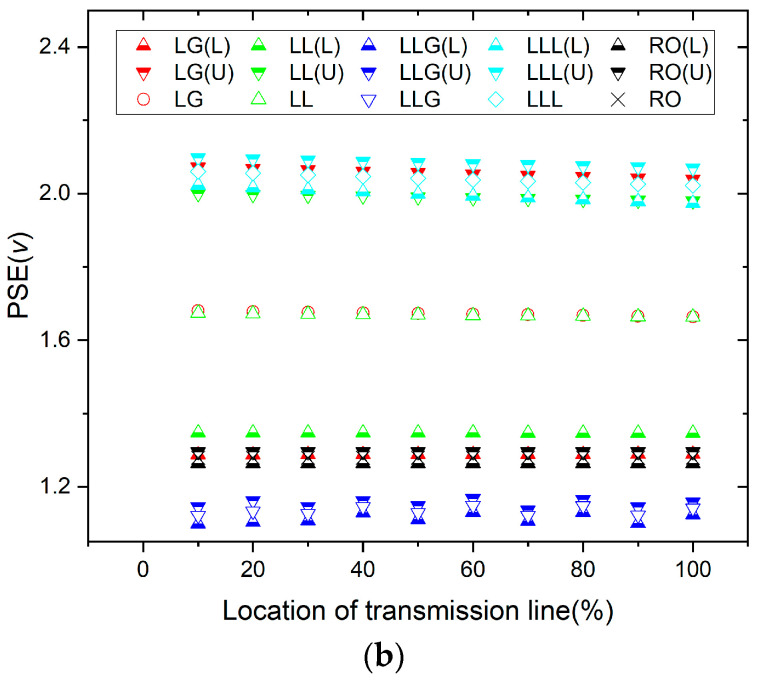
(**a**) Mean and error for the current without preprocessing. (**b**) Mean and error for the voltage without preprocessing.

The mean and error of the three-phase measurement values are used to compare the effects of data preprocessing and non-preprocessing. Without data preprocessing, the results of calculating the mean and error of the PSE’s value of the three-phase current and three-phase voltage at the fault point are shown in Figure 5a and Figure 5b, respectively. It can be seen from Figure 5a,b that the mean and error of regular operation are within the range of the fault. Identifying the state of the regular operation or malfunction through the mean value is difficult. In addition, if the judgment is based on the distance between the upper and lower bounds, there is a chance of interpretation error, and the regular operation state is judged as a fault state or vice versa. This result is likely to cause misjudgment of the relay protection device. 

**Figure 6:**
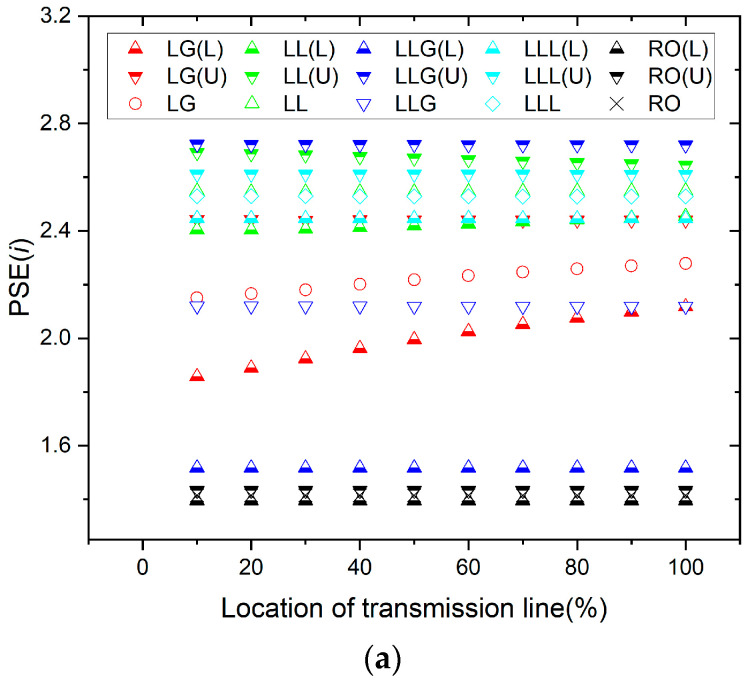

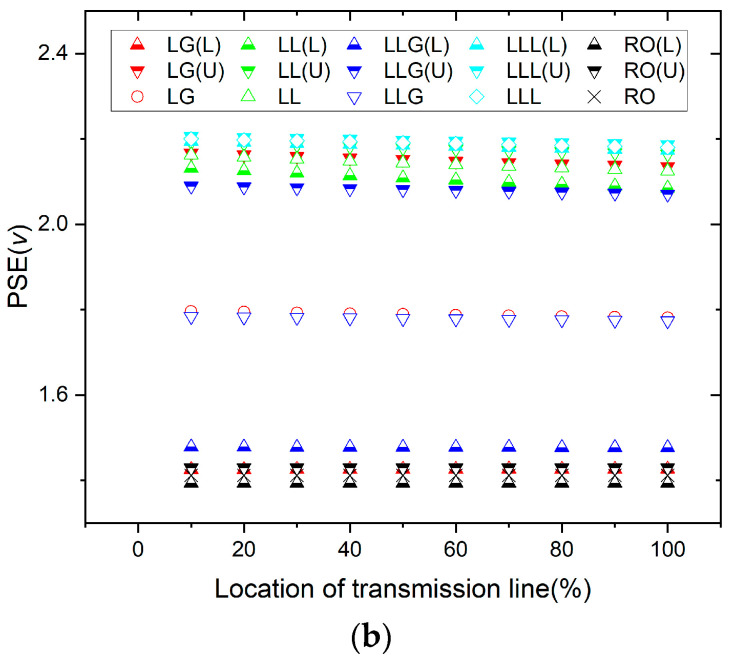
(**a**) PSE’s value with mean and error for the current. (**b**) PSE’s value with mean and error for the voltage.

With data preprocessing, the results of calculating the mean and error of the PSE’s value of the three-phase current and three-phase voltage at the fault point are shown in Figure 6a and Figure 6b, respectively. It can be seen from Figure 6a,b that the mean and error of regular operation are trend towards smaller values. The reason is that the normal signal does not have the asymmetric component that is additionally generated by the fault signal. If only the figure’s mean value were examined, it would clearly distinguish whether it is in regular operation or fault state. The mean value of the normal operation state is smaller than the mean value of all kinds of faults. In addition, if the interpretation is based on the distance between the upper and lower bounds, it is also clear that the distance between the upper and lower bounds for the fault is larger than the distance between the upper and lower bounds for the regular operation.

The results of Figure 5 and Figure 6 show that the data preprocessed by EEMD has better resolution, highlighting the benefits of combining the two methods. Data preprocessing alleviates the data sensitivity of the measurement. 

## 4. Discussion

The rules in Table 1 demonstrate that when the system experiences the LG fault, the current for the fault phase may reach more than ten to dozens of times the normal operating current. The voltage is also increased for the fault phase, and the voltage remains unchanged a lot in transient for the non-faulty phase but may be affected by other factors. The voltage for the fault-free phase is slightly reduced compared with the fault phase. Its fluctuations show that the value of the PSE (*i* and *v*) for the fault phase is increased and reached the maximum. The value of the PSE (*i* and *v*) for the fault-free phases also increases because of the harmonics generated during the fault.

From the rules in Table 2, one can see that for the case of the LL fault, the current for the fault phase may reach more than ten to dozens of times the normal operating current, and the voltage is equal to the pre-fault voltage for the fault-free phase. The voltage for the fault phase is only half of the voltage for the non-faulty phase, and the direction is opposite. The value of the PSE (*i* and *v*) for the fault phase is built up, but the increment value is higher than that of the fault-free phase. The value of the PSE (*i* and *v*) for the fault-free phases is also changed a little.

It can be seen from the rules in Table 3 that when the LLG fault occurs in the system, the current for the fault phase may reach more than ten times to dozens of times the normal operating current, and the voltage for the fault-free phase is lower than for the faulty phase a little. The value of the PSE (*i* and *v*) for the fault phase is extended, but the increment value is higher than that of the fault-free phase, and the value of the PSE (*i* and *v*) for the fault-free phase also advances.

It can be seen from the rules in Table 4 that for the situation of the LLL fault, the PSE (*i* and *v*) values for the fault phase are intensified. Therefore, the case of the LLL fault is identified by the value of the PSE (*i* and *v*) between the fault phase and the normal operating conditions.

When a short circuit occurs, the system’s voltage will drop significantly. The fault for current and voltage waveforms is more complicated than regular signals. The frequency components of fault signals are prosperous, which leads to an increase in the PSE value. Therefore, a closer short circuit distance causes the system’s current to become more complex and the PSE value to increase. Based on the above data, the fault is determined by the PSE value of the fault phase when the fault occurs at the transmission line.

In the power system, when the fault occurs on the transmission line, the closer the fault point, the smaller the PSE value of the current and voltage. The reason is that the increase in resistance near the fault point attenuates the signal magnitude. This attenuation causes the signal’s spectral region to become smaller, thereby reducing the PSE value. That is, near the fault point, the signal’s spectrum is more restricted, the spectrum range area has become smaller, and the PSE value is decreased. Therefore, the closer the fault point, the smaller the PSE (*i* and *v*) value is. Based on the above data, when the transmission line fault occurs, the fault location is determined by the value of the PSE of the faulty phase. The different values of the PSE (*i* and *v*) discover the spot for the symmetrical and asymmetrical faults for the fault phase. Thus, it can be seen that the PSE value is a valuable index for evaluating the fault location of the transmission line.

## 5. Conclusions

This paper proposes information measurement using the PSE to detect 110 kV transmission line fault points by analyzing the changing characteristics of the PSE value under regular operation and different types of faults and performing fault diagnosis based on the value of the PSE. Data preprocessing is introduced using the EEMD method, and similarity for data reconstruction is used to improve the capability for fault diagnosis. The results show that using the PSE value to extract transmission line fault signals has a high degree of identification of fault locations and fault types, which are used as characteristic indicators to characterize different states of transmission lines. Finally, this combined method was applied to an example of feature extraction from the simulation model discussed in the article. It was verified that the quantities of extracted features are distinguished by different states of faults and the fault location of the transmission line. Therefore, using the combined methods, the PSE value at the faulty phase is used as one of the auxiliary detection reference indicators to distinguish the fault type and location of the transmission line.

## Figures and Tables

**Table 1:** Results of LG fault.

PSE	*i_A_*	*i_B_*	*i_C_*	*v_A_*	*v_B_*	*v_C_*
10%	2.46468	1.88660	2.09875	2.22387	1.59409	1.57016
20%	2.46321	1.91518	2.11969	2.21987	1.59319	1.56947
30%	2.46224	1.95834	2.12055	2.21554	1.59228	1.56901
40%	2.46121	1.98945	2.15431	2.21150	1.59163	1.56854
50%	2.46029	2.02206	2.17150	2.20763	1.59080	1.56764
60%	2.45941	2.05131	2.18770	2.20382	1.59006	1.56720
70%	2.45858	2.07753	2.20272	2.20017	1.58961	1.56676
80%	2.45778	2.10094	2.21650	2.19641	1.58863	1.56616
90%	2.45702	2.12179	2.22905	2.19321	1.58793	1.56558
100%	2.45630	2.14043	2.24043	2.18952	1.58724	1.56534
RO	1.42620	1.39136	1.42516	1.39867	1.40357	1.43162

**Table 2:** Results of LL fault.

PSE	*i_A_*	*i_B_*	*i_C_*	*v_A_*	*v_B_*	*v_C_*
10%	2.63014	2.63275	2.38153	2.14312	2.19759	2.14543
20%	2.62584	2.62981	2.38130	2.13863	2.19416	2.13908
30%	2.62171	2.62702	2.38553	2.13424	2.19076	2.13324
40%	2.61779	2.62439	2.39203	2.12995	2.18758	2.12716
50%	2.61414	2.62185	2.39975	2.12557	2.18487	2.12131
60%	2.61064	2.61946	2.40797	2.12121	2.18209	2.11674
70%	2.60730	2.61720	2.41631	2.11747	2.17894	2.11127
80%	2.60412	2.61501	2.42458	2.11378	2.17626	2.10608
90%	2.60108	2.61291	2.43249	2.11025	2.17356	2.10163
100%	2.59817	2.61091	2.44001	2.10642	2.17044	2.09671
RO	1.42620	1.39136	1.42516	1.39867	1.40357	1.43162

**Table 3:** Results of LLG fault.

PSE	*i_A_*	*i_B_*	*i_C_*	*v_A_*	*v_B_*	*v_C_*
10%	2.47218	2.46368	1.42445	1.93754	1.98244	1.43166
20%	2.47196	2.46345	1.42444	1.93591	1.98043	1.43162
30%	2.47173	2.46322	1.42443	1.93435	1.97870	1.43164
40%	2.47151	2.46299	1.42442	1.93278	1.97732	1.43168
50%	2.47128	2.46276	1.42440	1.93139	1.97517	1.43169
60%	2.47106	2.46254	1.42440	1.92984	1.97354	1.43166
70%	2.47084	2.46232	1.42439	1.92828	1.97158	1.43175
80%	2.47061	2.46209	1.42439	1.92662	1.96983	1.43167
90%	2.47038	2.46187	1.42438	1.92517	1.96805	1.43167
100%	2.47014	2.46164	1.42437	1.92360	1.96601	1.43164
RO	1.42620	1.39136	1.42516	1.39867	1.40357	1.43162

**Table 4:** Results of LLL fault.

PSE	*i_A_*	*i_B_*	*i_C_*	*v_A_*	*v_B_*	*v_C_*
10%	2.43759	2.55454	2.59627	2.19564	2.19793	2.20611
20%	2.43744	2.55432	2.59616	2.19327	2.19553	2.20389
30%	2.43731	2.55411	2.59605	2.19085	2.19335	2.20176
40%	2.43717	2.55388	2.59594	2.18862	2.19149	2.19966
50%	2.43701	2.55368	2.59584	2.18638	2.18901	2.19778
60%	2.43687	2.55346	2.59575	2.18405	2.18708	2.19566
70%	2.43671	2.55323	2.59564	2.18174	2.18456	2.19362
80%	2.43656	2.55300	2.59555	2.17934	2.18218	2.19150
90%	2.43643	2.55279	2.59545	2.17732	2.17973	2.18954
100%	2.43629	2.55257	2.59534	2.17516	2.17741	2.18740
RO	1.42620	1.39136	1.42516	1.39867	1.40357	1.43162

## Data Availability

The original contributions presented in the study are included in the article, further inquiries can be directed to the corresponding author.
